# Thirty‐day hospital readmission rate, reasons, and risk factors after acute inpatient cancer rehabilitation

**DOI:** 10.1002/cam4.4154

**Published:** 2021-07-27

**Authors:** Jegy M. Tennison, Nahid J. Rianon, Joanna G. Manzano, Mark F. Munsell, Marina C. George, Eduardo Bruera

**Affiliations:** ^1^ Department of Palliative, Rehabilitation and Integrative Medicine The University of Texas MD Anderson Cancer Center Houston TX USA; ^2^ Department of Family & Community Medicine and Joan & Stanford Alexander Division of Geriatric and Palliative Medicine McGovern Medical School The University of Texas Houston Health Science Center Houston TX USA; ^3^ Department of Hospital Medicine The University of Texas MD Anderson Cancer Center Houston TX USA; ^4^ Department of Biostatistics The University of Texas MD Anderson Cancer Center Houston TX USA

**Keywords:** cancer, continuity, hospital, inpatient, readmission, rehabilitation, thirty

## Abstract

**Objectives:**

To evaluate the 30‐day hospital readmission rate, reasons, and risk factors for patients with cancer who were discharged to home setting after acute inpatient rehabilitation.

**Design, Setting, and Participants:**

This was a secondary retrospective analysis of participants in a completed prospective survey study that assessed the continuity of care and functional safety concerns upon discharge and 30 days after discharge in adults. Patients were enrolled from September 5, 2018, to February 7, 2020, at a large academic quaternary cancer center with National Cancer Institute Comprehensive Cancer Center designation.

**Main Outcomes and Measures:**

Thirty‐day hospital readmission rate, descriptive summary of reasons for readmissions, and statistical analyses of risk factors related to readmission.

**Results:**

Fifty‐five (21%) of the 257 patients were readmitted to hospital within 30 days of discharge from acute inpatient rehabilitation. The reasons for readmissions were infection (20, 7.8%), neoplasm (9, 3.5%), neurological (7, 2.7%), gastrointestinal disorder (6, 2.3%), renal failure (3, 1.1%), acute coronary syndrome (3, 1.1%), heart failure (1, 0.4%), fracture (1, 0.4%), hematuria (1, 0.4%), wound (1, 0.4%), nephrolithiasis (1, 0.4%), hypervolemia (1, 0.4%), and pain (1, 0.4%). Multivariate logistic regression modeling indicated that having a lower locomotion score (OR = 1.29; 95% CI, 1.07–1.56; *p* = 0.007) at discharge, having an increased number of medications (OR = 1.12; 95% CI, 1.01–1.25; *p* = 0.028) at discharge, and having a lower hemoglobin at discharge (OR = 1.31; 95% CI, 1.03–1.66; *p* = 0.031) were independently associated with 30‐day readmission.

**Conclusion and Relevance:**

Among adult patients with cancer discharged to home setting after acute inpatient rehabilitation, the 30‐day readmission rate of 21% was higher than that reported for other rehabilitation populations but within the range reported for patients with cancer who did not undergo acute inpatient rehabilitation.

## Short Abstract

Research is needed to determine the rates of and risk factors for 30‐day hospital readmission after acute inpatient cancer rehabilitation to understand the nature of this problem and to decrease costs associated with readmissions. Among adult patients with cancer discharged to a home setting after acute inpatient rehabilitation, the 30‐day readmission rate of 21% was higher than that reported for other rehabilitation populations but within the range reported for patients with cancer who did not undergo acute inpatient rehabilitation. Cancer rehabilitation patients may have unique risk factors for readmission.

## 1 INTRODUCTION

Readmission to an acute‐care hospital is considered to be an indicator of the quality of care.^1^ The expense of unplanned readmissions is 15 to 20 billion dollars annually,^2^, ^3^ so reducing the hospital readmissions rate (HRR) is a top priority of U.S. health care reform efforts. The U.S. government created the Hospital Readmission and Reduction Program to financially penalize hospitals with above‐average readmission rates for Medicare patients.^2^, ^3^, ^4^ These penalties for readmissions do not apply to cancer hospitals because the needs of patients with cancer are challenging to compare to those of other hospitalized patients.^3^ Cancer care costs are, however, expected to continue to dramatically increase because of the growing demand for services and the increasing complexity of cancer treatment.^5^ Thus, decreasing the 30‐day HRR could reduce health care costs, improve health care quality,^6^, ^7^ and enhance the patient experience.^7^ It could also specifically change outcomes for patients with advanced cancer, who have a high rate of health care utilization due to hospitalization and therefore have been recommended to reduce reliance on acute hospital care and to increase access to palliative care to be consistent with patient‐centered care.^8^, ^9^ Patients with advanced cancer have been reported to have worse quality of life if dying in a hospital^10^ and may prefer home as the place of terminal care.^11^, ^12^

Since Medicare initiated the inpatient prospective payment system in 1983, the U.S. hospitals have tried to find ways to reduce hospital length of stay in part by increasing the use of post‐acute care facilities such as inpatient rehabilitation facilities (IRFs), long‐term acute care hospitals (LTACHs), and skilled nursing facilities (SNFs).^13^ Discharging specifically to LTACHs and SNFs have been found to be associated with higher likelihood of readmission or death.^14^

The Centers for Medicare & Medicaid Services have also identified 30‐day HRR as a national quality indicator for IRFs,^15^ which can be freestanding or units within acute‐care hospitals.^1^ Acute inpatient rehabilitation at IRFs serves a critical role in the continuum of care^6^ by providing patients with simultaneous medical and rehabilitative care to significantly improve their functional status so that they can be safely discharged, ideally to their home setting.^16^ Acute inpatient rehabilitation differs from inpatient rehabilitation at LTACHs and SNFs in that patients are mandated by payors to participate in an intensive 3 hours of rehabilitation per day at least 5 days of the week and are required to be supervised by a rehabilitation physician with face‐to‐face visits at least 3 days a week with a coordinated, interdisciplinary team approach.^17^ Patients with cancer are categorized as “medically complex conditions,” which is one of the rehabilitation impairment categories for inpatient rehabilitation and includes a range of cancer‐ and noncancer‐related diagnoses.^18^ Fisher et al.^19^ showed that medically complex patients’ functional status and length of rehabilitation stay were the best predictors of 30‐day rehospitalization. While many studies have evaluated 30‐day HRR after acute inpatient rehabilitation^20^, ^21^, ^22^, ^23^, ^24^ for a diverse mix of rehabilitation impairment groups,^18^ none of these studies evaluated 30‐day HRR solely in patients with cancer who have undergone acute inpatient rehabilitation. One systematic review evaluating the 30‐day HRR in patients with cancer showed a wide range of rates from less than 3% to 34%.^7^ Indeed, patients with cancer have several demographic and clinical risk factors for rehospitalization.^7^, ^25^, ^26^, ^27^, ^28^, ^29^ There is, however, a gap in knowledge regarding 30‐day HRR and its associated risk factors purely in the cancer rehabilitation population who underwent acute inpatient rehabilitation.

The purpose of this study was to determine the 30‐day HRR and the reasons and risk factors for rehospitalization among patients with cancer who were discharged to their home setting after acute inpatient rehabilitation. A better understanding of the frequency of and reasons for rehospitalization in this patient population and identifying their unique risk factors will help improve care planning for this patient population. It could also help develop interventions to avoid preventable readmissions and improve patient‐centered outcomes to be consistent with patient's preference to be home for terminal care in patients with advanced cancer.

## 2 METHODS

### 2.1 Participants

All consecutive patients who were admitted to acute inpatient rehabilitation service from within our hospital between September 5, 2018 and February 7, 2020 were screened for eligibility for a survey study assessing continuity of care and functional safety concerns upon discharge (first survey) and 1 month after discharge (second survey).^30^ For this study, a secondary retrospective analysis was performed using data for all patients who participated in the survey study. Thus, the following inclusion criteria were used: patients had to (a) have been discharged from the acute cancer inpatient rehabilitation unit to their home setting; (b) provide informed consent; (c) be 18 years of age or older; and (d) be English‐speaking. The exclusion criteria were: (a) discharge to another hospital or health facility; (b) moderate to severe cognitive deficits; and (c) readmission to the acute inpatient rehabilitation service after the completion of both surveys. These inclusion and exclusion criteria were applied because the aims of this study were added to the original prospective survey study protocol. For this study, we also excluded four patients who died within 1 month after discharge. Additional exclusion criteria used for the original prospective survey study but not applied to this study were: (a) rehospitalization at any time during the study period and (b) not reachable via telephone after three attempts to complete the repeated survey 1 month after discharge. Thus, all patients who enrolled in the survey study, including those who were not able to complete the second survey 30 days after discharge, were included in this study.

### 2.2 Design and data collection

Approval for data collection was obtained from our Institutional Review Board. Study data were managed using the Research Electronic Data Capture tool.

Patients’ demographic, clinical, and functional characteristics (see Table 1) were collected retrospectively from the institution's electronic health record by research staff and by the principal investigator. These variables were analyzed as described in the Statistical Analyses section to ascertain risk factors for 30‐day hospital readmissions.

**TABLE 1:** Characteristics of patients who underwent acute inpatient cancer rehabilitation

		Readmission within 30 days		
Characteristic, No. (%)	Total (N = 257)	No (N = 202)	Yes (N = 55)	*p*‐value
Female	106 (41)	86 (43)	20 (36)	0.44^a^
Race				0.18^a^
White	214 (83)	167 (83)	47 (85)	
Black	22 (9)	20 (10)	2 (4)	
Asian	9 (4)	5 (2)	4 (7)	
Other	12 (5)	10 (5)	2 (4)	
Hispanic ethnicity	24 (9)	18 (9)	6 (11)	0.84^a^
Married / significant other	167 (65)	136 (67)	31 (56)	0.15^a^
Cancer diagnosis				0.15^b^
Hematologic & lymphatic	63 (25)	44 (22)	19 (35)	
Brain & other nervous system	54 (21)	49 (24)	5 (9)	
Breast	10 (4)	8 (4)	2 (4)	
Digestive	18 (7)	12 (6)	6 (11)	
Bones and joints	39 (15)	33 (16)	6 (11)	
Respiratory	17 (7)	12 (6)	5 (9)	
Genitourinary	34 (13)	27 (13)	7 (13)	
Other^c^	22 (9)	17 (8)	5 (9)	
No surgery during hospitalization	101 (39)	70 (35)	31 (56)	***0.005*** ^a^
**Characteristic, median (IQR)**				
Age, years	63 (51, 70)	64 (51,70)	60 (50, 68)	0.41^d^
Hospital length of stay, days	23 (17, 31)	23 (17, 30)	24 (17, 36)	0.27^d^
Rehabilitation stay, days	11 (8, 14)	11 (8, 14)	10 (8, 14)	0.33^d^
No. of admissions in past 12 months	1 (0, 2)	1 (0, 2)	1 (0, 3)	***0.04*** ^d^
Admission FIM^e^—transfer (bed, chair)	4 (4, 4)	4 (4, 4)	4 (4, 5)	0.36^d^
Discharge FIM ‐ transfer (bed, chair)	5 (4, 6)	5 (5, 6)	5 (4, 6)	0.69^d^
Admission FIM ‐ locomotion (gait)	4 (1, 4)	4 (1, 4)	4 (1, 4)	0.49^d^
Discharge FIM – locomotion (gait)	5 (4, 5)	5 (4, 5)	4 (2, 5)	***0.003*** ^d^
Elixhauser medical comorbidity index	3 (2, 5)	3 (2, 4)	4 (2, 5)	0.19^d^
Discharge medications, total	9 (7, 11)	9 (7, 11)	9 (8, 12)	***0.009*** ^d^
Laboratory values, discharge^f^				
White blood cell (Ref: 4–11 K/μL)	5.6 (3.9, 7.6)	5.7 (4.1, 7.9)	5.0 (3.3, 7.1)	0.10^d^
Hemoglobin (Ref: 12–16 g/dL)	9.9 (9.1, 11.1)	10.2 (9.1, 11.3)	9.6 (8.9, 10.7)	***0.02*** ^d^
Platelet (Ref:140–440 K/μL)	195 (118, 279)	207 (124, 289)	165 (93, 269)	0.07^d^
Creatinine (Ref: 0.60–1.00 mg/dL)	0.76 (0.63, 0.97)	0.75 (0.63, 0.95)	0.82 (0.60, 1.06)	0.31^d^
Sodium (Ref: 135–147 mEq/L)	139 (136, 140)	139 (136, 140)	138 (136, 140)	0.75^d^

^a^ Fisher exact test.

^b^ Chi‐square test.

^c^ Other neoplasms include endocrine, oral cavity and pharynx, skin, other soft tissue, and unknown.

^d^ Wilcoxon rank‐sum test.

^e^ Functional Independence Measure.

^f^ Laboratory values were obtained 0–3 days before discharge and closest to the discharge day.

We used functional independence measure (FIM) scores, which were completed by physical therapists, to assess locomotion, and bed/chair transfer skills. The FIM instrument is the most widely used global functional assessment tool^31^ and has reliability greater than 0.85.^32^ Each item is scored from 1 to 7, with a higher score indicating more independence and a lower score indicating more dependence; a score of 1 indicates that the patient requires total assistance from a helper, while a score of 7 indicates that the patient is completely independent and requires no helper. We used scores for 2 of the 13 motor items on the FIM instrument that were readily available in our electronic health records: bed/chair transfer and walk/wheelchair locomotion skills. We did not use the other items on the FIM instrument because these were not consistently recorded within the recommended time frame of 72 hours after admission and 72 hours before discharge.

The outcome of interest was 30‐day HRR, which was calculated by dividing the number of patients who were readmitted within 30 days after discharge from acute inpatient rehabilitation by the total number of patients who enrolled in the study upon discharge from acute inpatient rehabilitation. The primary reason for readmission was identified based on the electronic health record. We did not differentiate between planned and unplanned readmissions for consistency with other studies of rehabilitation patients.^6^, ^21^ Usually, patients at our institution who are planned to undergo additional intensive oncologic treatment transfer back to acute‐care oncology services to receive their treatment (e.g., chemotherapy, surgery).

### 2.3 Statistical analyses

Descriptive statistics were used to summarize demographic and clinical characteristics overall and separately for patients who were readmitted to the hospital within 30 days of discharge from inpatient rehabilitation service and those who were not. Categorical variables were summarized with counts and percentages, and the Fisher exact test or the chi‐square test were used to test for associations between these characteristics and hospital readmission. Continuous variables were summarized with medians and interquartile ranges. The Wilcoxon rank‐sum test was used to compare the medians of continuous variables.

For those characteristics with a *p*‐value of less than 0.05, logistic regression methods were used to model the association between these potential risk factors and hospital readmission. The association is reported as an odds ratio (OR) and its 95% CI. Stepwise regression methods were then used to fit a multivariate model for the association between these risk factors and hospital readmission. Only those factors with a *p*‐value of less than 0.05 were included in the final model. All statistical analyses were performed using SAS 9.4 (SAS Institute, Inc.). The primary reasons for hospital readmission were tabulated and summarized with counts and percentages.

## 3 RESULTS

**FIGURE 1:**
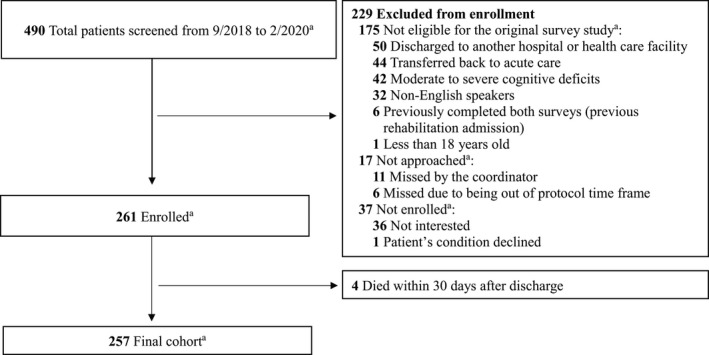
Cohort selection of patients who underwent acute inpatient rehabilitation. ^a^This study is a secondary retrospective analysis of participants in a completed survey study

Among the 490 total patients screened for enrollment in the original survey study, 229 patients were excluded (see Figure 1). These patients were excluded because of ineligibility with the original survey study (175 patients), some were missed by the research coordinator (17 patients), and some did not enroll (37 patients). Among the remaining 261 patients who were discharged to their home setting, 55 (21%) patients were readmitted and 4 (2%) died within 30 days. Most of the patients were men (59%), white (83%), and married (65%). Their median age was 63 years, and the median length of hospital stay, which included the length of the acute inpatient rehabilitation stay, was 23 days. The most common type of primary neoplasm was hematologic and lymphatic neoplasms (25%).

Table 1 compares the characteristics of the patients who did and did not have 30‐day readmissions. The characteristics with a *p*‐value of less than 0.05 were used to evaluate the association between these potential risk factors and hospital readmission (Table 2). The univariate analysis showed that the following factors were associated with 30‐day readmission: no surgery during hospitalization (*p* = 0.004), decreased locomotion scores upon discharge (*p* = 0.004), increased the total number of discharge medications (*p* = 0.003), and decreased hemoglobin level at discharge (*p* = 0.015). We eliminated further analysis of the medical admissions variable (no surgery during hospitalization) since it is intuitive that surgical admissions are usually curative and are less likely to be readmitted compared to medical admission patients, who have been reported to have higher rate of readmissions.^7^, ^33^

**TABLE 2:** Logistic regression analyses of characteristics related to 30‐day readmission among patients who underwent acute inpatient cancer rehabilitation

Characteristic	Univariate logistic regression			Multivariate logistic regression		
	OR^a^	95% CI	*p*‐value	OR	95% CI	*p*‐value
No. of admissions in past 12 months (increase of 1 unit)	1.20	0.98, 1.45	0.07	—	—	—
Discharge FIM^b^ – locomotion (decrease of 1 unit)	1.30	1.08, 1.55	0.004	1.29	1.07, 1.56	0.007
Discharged medications, total (increase of 1 unit)	1.15	1.05, 1.26	0.003	1.12	1.01, 1.25	0.028
Discharge hemoglobin (g/dL) (decrease in 1 unit)	1.32	1.06, 1.66	0.015	1.31	1.03, 1.66	0.031

^a^ OR, odds ratio.

^b^ FIM, functional independence measure.

In the multivariate analysis, decreased locomotion score upon discharge (*p* = 0.007), increased total medications at discharge (*p* = 0.028), and decreased hemoglobin at discharge (*p* = 0.031) remained significantly and independently associated with readmission. For discharge locomotion score, the OR of 1.29 indicated that the odds of hospital readmission increase by 1.29 for every unit decrease in this measure. Another factor found to be statistically significant at the 0.05 level in the multivariate model was total medications at discharge, with an OR of 1.12, indicating that the odds of hospital readmission increased by 1.12 for each additional medication prescribed at discharge. Last, the OR for hemoglobin at discharge was 1.31, suggesting that the odds of hospital readmission increased by 1.31 for each unit decrease in hemoglobin.

The most common reason for readmission was infection (20/257, 7.8%) and the median number of days to readmission was 14 (range, 7–21) for the entire cohort (see Table 3). Of the 55 readmitted patients, 4 were admitted to a hospital other than our institution.

**TABLE 3:** Primary reason for readmission after acute inpatient cancer rehabilitation

Reason for readmission	No. of patients (%) N = 257
All‐cause readmissions	55 (21)
Infection	20 (7.8)
Neoplasm	9 (3.5)
Neurological (focal deficits, seizures)	7 (2.7)
Gastrointestinal disorder	6 (2.3)
Renal failure	3 (1.2)
Acute coronary syndrome	3 (1.2)
Others^a^	7 (2.7)
Number of days to readmission, median (IQR 25–75)	14 (7–21)

^a^ Other reasons included heart failure, fracture, hematuria, wound, nephrolithiasis, hypervolemia, and pain.

## 4 DISCUSSION

We found a 30‐day HRR of 21% in this cohort of patients who were discharged to their home setting after undergoing acute inpatient cancer rehabilitation at a large academic quaternary cancer center. Our result is within the range of HRRs (< 3% to 34%) reported in a systematic review assessing readmission rates for patients with cancer discharged from hospitals.^7^ Our readmission rate of 21% is, however, greater than the range of 5.8% to 18.8% reported for other types of inpatient rehabilitation patients (stroke, brain dysfunction, lower extremity fracture, lower extremity joint replacement, neurologic disorders, debility, etc.).^21^ This could be because some patients with cancer (medical more than surgical^33^) may be at higher risk for readmission.^28^ At our institution, we have noted that the majority of acute inpatient cancer rehabilitation patients (96%) have one or more medical complications; these complications can affect multiple organ systems.^16^ Interestingly, we have also previously noted the same rate (21%) of planned and unplanned transfer back to acute‐care oncology services.^16^ Furthermore, inadequate continuities of care after hospital discharge have often been noted in patients with cancer, who thus require closer coordination of care.^34^

In our cohort, the most common reason for readmission was infection, which has been reported to be one of the more common reasons for readmission in cancer patients^7^ as well as in other patients discharged from acute inpatient rehabilitation.^21^ Infection is also a common reason for returning to acute‐care oncology services from acute inpatient cancer rehabilitation.^16^ The median time to readmission was 14 days in this study, which is close to the median of 10 to 13 days reported in the literature for patients with cancer.^25^, ^28^, ^33^

The three risk factors that were found to be significantly and independently associated with readmission were decreased locomotion scores at discharge, increased the total number of medications at discharge, and decreased hemoglobin at discharge. We analyzed functional status using the FIM bed/chair transfer and walk/wheelchair locomotion scores and found that lower walk/wheelchair locomotion score at discharge was independently associated with readmission. This is consistent with a study showing that lower motor function scores were associated with readmission in medically complex rehabilitation patients.^19^ An increased total number of medications and decreased hemoglobin at discharge have been previously found to be independently associated with 30‐day readmission in patients with cancer.^28^ These risk factors may not be modifiable but they may identify patients who may benefit from intensive interventions during the transition of care period.^28^ Since polypharmacy (≥8 medications) has been associated with physical function impairment in older adults with cancer,^35^ patients with these characteristics may benefit from closer follow‐up assessments and in ensuring access to palliative care to apply patient‐centered care.

We did not assess socioeconomic factors because these data were not readily available. We did not find significant associations between readmission and demographic factors,^7^, ^21^ length of stay,^7^, ^21^ or comorbidities.^7^, ^25^, ^28^ Research with a larger sample size without this study's exclusion criteria may be useful to further evaluate these factors. It is also possible that the cancer rehabilitation population has unique risk factors for readmission. Medically stable patients are selected for acute inpatient rehabilitation; so that these patients can participate in and have meaningful, measurable functional improvements during the intensive rehabilitation program without medical instability interruptions.^16^ This may explain why comorbidities were not found to be significantly associated with readmission in our cohort.

### 4.1 Limitations

There are several limitations to this retrospective and relatively small study from a single institution. About half of the screened patients could not be enrolled. Retrospective studies by nature depend on the accuracy and completeness of the data available through the electronic health records. This study's findings are from a quaternary cancer center, and this setting differs from other conventional acute inpatient rehabilitation services or facilities, where there would be a different case mix. Thus, the results may not be generalizable to other, less‐specialized settings. We are also not able to compare oncological staging in this heterogeneous mix of cancer types since each blood cancer (considered to be intrinsically “metastatic”) has its own unique staging system compared to most solid tumors which use the TNM (tumor, node, metastasis) staging system.

This study also has limitations related to the exclusion criteria (see Figure 1 for details) used for the original survey study; thus, selection bias may be applicable. Patients with moderate to severe cognitive deficits could be a group with a higher risk of readmission and should be included in future studies of readmissions. Of note, it has been suggested that patients who were transferred to another health facility should be excluded because the risk factors for readmission should be measured at the time of discharge from the discharging facility and based on the discharging facility's data.^28^ We excluded these patients because of the original survey study's exclusion criteria.

We also excluded those patients who were directly transferred back to acute care oncology services from acute inpatient rehabilitation unless the patients returned to complete acute inpatient rehabilitation and were discharged to home. Some of these transfers were for planned treatments and we did not differentiate between planned and unplanned readmissions for consistency with other studies of rehabilitation patients^6^, ^21^ (which would include cancer rehabilitation population as a minority among a mix of heterogeneous mix of rehabilitation patients).

To the best of our knowledge, this is the first study to report the 30‐day HRR after acute inpatient cancer rehabilitation, and thus the data are valuable as an overall finding for the cancer rehabilitation population. This analysis of readmission rates, reasons for readmission, and risk factors for readmission provides data with which to initiate efforts to improve transitions of care, reduce costs, and improve health care quality for this population. Future studies should address the aforementioned limitations to better capture and validate readmission rates, reasons, and risk factors for the cancer rehabilitation population.

## 5 CONCLUSIONS

We report a higher 30‐day HRR in our cohort of cancer rehabilitation patients compared to other rehabilitation patients. Cancer rehabilitation patients may have unique risk factors for readmission. While decreased hemoglobin level at discharge, decreased locomotion scores at discharge, and increased the total number of medications at discharge were independently associated with 30‐day HRR in our study, we recommend a future longitudinal study without our study's limitations to further validate the risk factors associated with the cancer rehabilitation population.

## DISCLOSURES

Supported in part by the National Institutes of Health/National Cancer Institute under Cancer Center Support Grant P30CA016672; used the Biostatistics Shared Resource.

The preliminary findings were presented at the joint Association of Academic Physiatrists Annual Meeting and International Society of Physical and Rehabilitation Medicine, in Orlando, Florida on March 9, 2020.

## CONFLICT OF INTERESTS

The authors declare no potential conflicts of interest

## Data Availability

The data that support the findings of this study are available from the corresponding author upon reasonable request.
